# Evaluation of sleep quality and obstructive sleep apnea in non-alcoholic fatty liver disease: a cross-sectional study

**DOI:** 10.1186/s12876-026-05298-z

**Published:** 2026-09-02

**Authors:** Sahar Ravanshad, Mohadese Nikookar Golestani, Mitra Ahadi, Mona Kabiri, Hassan Mehrad-Majd, Lahya Afshari Saleh, Anahita Azinfar, Mehrshad Rajabi

**Affiliations:** 1 Department of Internal Medicine, Faculty of Medicine, Mashhad University of Medical Sciences, Mashhad, Iran; 2 Department of Gastroenterology and Hepatology, Mashhad University of Medical Sciences, Mashhad, Iran; 3 Nanotechnology Research Center, Pharmaceutical Technology Institute, Mashhad University of Medical Sciences, Mashhad, Iran; 4 Department of Pharmaceutical Nanotechnology, School of Pharmacy, Mashhad University of Medical Sciences, Mashhad, Iran; 5 Clinical Research Development Unit, Mashhad University of Medical Sciences, Mashhad, Iran; 6 Department of Occupational Medicine, Faculty of Medicine, Mashhad University of Medical Science, Mashhad, Iran

**Keywords:** Non-alcoholic fatty liver disease, Sleep quality, Pittsburgh Sleep Quality Index, Obstructive sleep apnea, STOP-BANG

## Abstract

**Background:**

Sleep is essential for metabolic homeostasis and overall health. NAFLD, recently reclassified as MASLD, is the most prevalent chronic liver disease globally (~ 25% prevalence) and is strongly linked to metabolic comorbidities. Growing evidence associates adverse sleep conditions including short sleep duration, OSA, and circadian misalignment with NAFLD onset and progression. However, data from Iranian and Middle Eastern populations remain scarce, and no study has simultaneously evaluated sleep quality and OSA risk in a large Iranian NAFLD cohort. This study aimed to address this gap using the PSQI and STOP-BANG questionnaire.

**Methods:**

This cross-sectional study utilized registry data from the PERSIAN Organizational Cohort Study (POCS) at Mashhad University of Medical Sciences. A total of 958 NAFLD patients diagnosed by abdominal ultrasonography were included.

**Results:**

Among 958 participants (69.6% male; mean age 46.7 ± 9.1 years), 35.4% had sleep disturbance (31.6% mild; 3.8% moderate). Over half (53.4%) were at intermediate OSA risk. Sleep disturbance was significantly associated with female sex (*p* = 0.022), single marital status (*p* = 0.019), higher BMI (*p* = 0.026), and hypertension (*p* = 0.007). BMI correlated weakly but significantly with PSQI scores (*r* = 0.072, *p* = 0.036). Hepatic steatosis grade was not associated with sleep disturbance (*p* = 0.193) but correlated positively with STOP-BANG scores (*r* = 0.232, *p* < 0.001).

**Conclusions:**

Sleep disturbance and elevated OSA risk are common among patients with NAFLD and were associated, in unadjusted analyses, with female sex, elevated BMI, hypertension and single marital status. These findings support consideration of sleep assessment in this population on case-finding grounds; whether identifying or treating these conditions improves hepatic or metabolic outcomes cannot be determined from a cross-sectional study.

**Supplementary Information:**

The online version contains supplementary material available at https://doi.org/10.1186/s12876-026-05298-z.

## Background

Sleep is a fundamental biological process essential for metabolic homeostasis, cognitive function, immune regulation, and overall quality of life. In contemporary societies, sleep insufficiency has emerged as a growing public health concern, with millions of individuals worldwide failing to obtain adequate sleep to support healthy physiological function [1].

One disease closely linked to metabolic dysfunction is non-alcoholic fatty liver disease (NAFLD), recently renamed metabolic dysfunction-associated steatotic liver disease (MASLD). MASLD is defined as steatotic liver disease occurring alongside one or more cardiometabolic risk factors in the absence of significant alcohol consumption, and encompasses a spectrum ranging from simple steatosis and metabolic dysfunction-associated steatohepatitis (MASH) to fibrosis, cirrhosis, and ultimately MASH-related hepatocellular carcinoma [2].

NAFLD represents the most common form of chronic hepatic disease globally, with an estimated prevalence of approximately 25% of the world’s population [3] and an annual incremental rise of 0.7% according to recent analyses [4].

Beyond the intrahepatic complications including liver cirrhosis, hepatic decompensation, and hepatocellular carcinoma [5], NAFLD is associated with a broad spectrum of extrahepatic comorbidities. More than 70% of individuals with type 2 diabetes have NAFLD [6], and NAFLD independently increases the risk of cardiovascular morbidity and mortality [7], as well as chronic kidney disease [8].

Several sleep-related conditions have been implicated in the pathogenesis of NAFLD. A 2023 meta-analysis of fifteen studies comprising 261,554 participants revealed a robust positive association between short sleep duration and increased NAFLD risk in the pooled analysis [9]. Concurrently, a compelling body of evidence has demonstrated a strong relationship between NAFLD/MASLD and obstructive sleep apnea (OSA) [10]. Moreover, circadian misalignment arising from extended or prolonged night shift work (NSW) has been progressively linked to hepatic steatosis, impaired liver function, and the advancement of inflammatory liver disease, particularly NAFLD/MASLD, alongside an increased susceptibility to hepatocellular carcinoma [11].

Growing evidence suggests that sleep quality is impaired in patients with NAFLD. Using the Pittsburgh Sleep Quality Index (PSQI), Takahashi et al. demonstrated in 4,828 participants that daytime dysfunction was significantly higher in NAFLD patients compared to non-NAFLD individuals, with notable sex-specific differences [12]. Similarly, Marin-Alejandre et al. reported a significantly higher prevalence of poor sleep quality in obese NAFLD patients compared to healthy controls, and showed that sleep quality scores predicted up to 20% of the variability in liver stiffness, suggesting that sleep disruption may both contribute to and result from NAFLD progression [13].

Despite these findings, data from Iranian and Middle Eastern populations remain very limited. To date, only one small Iranian study has examined sleep quality using PSQI specifically in overweight NAFLD patients (*n* = 72), and no significant independent association was found after adjusting for BMI and age [14]. The small sample size, case-control design, and limited scope of that study restrict the generalizability of its findings to the broader Iranian NAFLD population. Furthermore, no study has simultaneously evaluated both sleep quality and obstructive sleep apnea risk in a large Iranian clinical cohort.

Therefore, the present cross-sectional study aimed to evaluate sleep quality using the PSQI and obstructive sleep apnea risk using the STOP-BANG questionnaire in a large cohort of NAFLD patients from the Persian Cohort of Mashhad University of Medical Sciences.

## Methods

This cross-sectional study was conducted using data from the PERSIAN Organizational Cohort Study (POCS) at Mashhad University of Medical Sciences (MUMS), a prospective population-based cohort designed to investigate the determinants of non-communicable diseases in Iranian adults. The cohort recruited participants aged 30–70 years and collected comprehensive baseline data including abdominal ultrasonography, anthropometric measurements, biochemical profiles, and validated questionnaires [15].

The study population comprised all participants of the POCS diagnosed with NAFLD based on ultrasonographic findings who had completed at least one of the Pittsburgh Sleep Quality Index (PSQI) and the STOP-BANG questionnaire. Hepatic steatosis was graded by abdominal ultrasonography as grade 1 (mild: slightly increased echogenicity with normal visualization of the diaphragm and the intrahepatic vessels), grade 2 (moderate: moderately increased echogenicity with slightly impaired visualization of the diaphragm or intrahepatic vessels), and grade 3 (severe: markedly increased echogenicity with poor or no visualization of the diaphragm or intrahepatic vessels) [15]. Baseline enrolment for the POCS cohort began following ethics approval in January 2017 and was completed in mid-2021 [15]; the present analysis uses baseline data from that period, extracted from the cohort registry on 4 July 2023.

Participants were excluded if they had: [1] significant alcohol consumption; [2] nightly use of sedative or hypnotic medications; [3] a prior diagnosis of obesity hypoventilation syndrome (OHS); [4] a prior diagnosis of depression or anxiety disorder; [5] Participants with missing data on key variables.

**Fig. 1:**
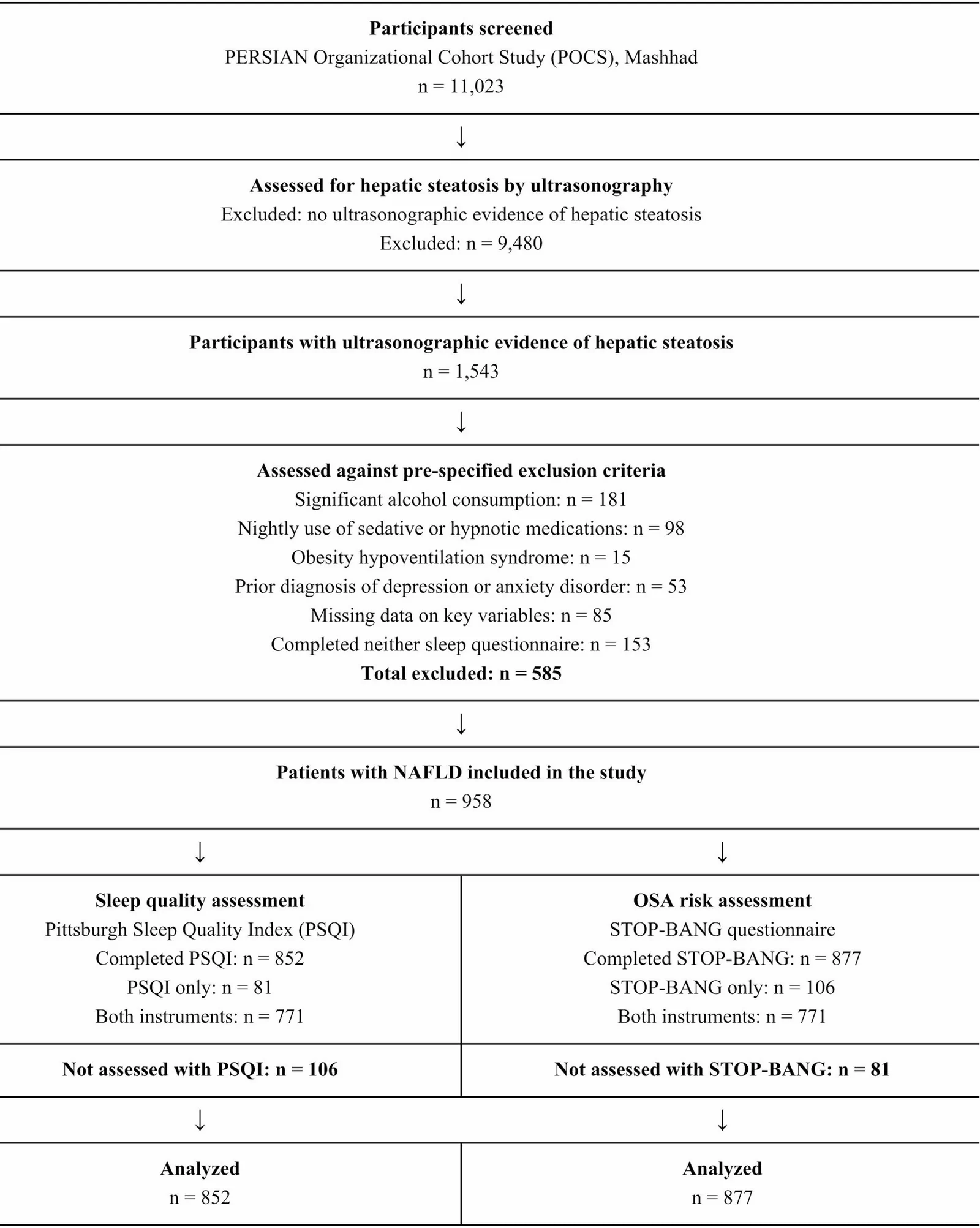
Flow of participants through the study, reported in accordance with the STROBE statement for cross-sectional studies. The two analysis sets are not independent: 771 participants completed both instruments, 81 completed the PSQI alone, and 106 completed the STOP-BANG alone. Analyses were conducted on an available-case basis

As shown in Fig. 1 771 participants completed both instruments, 81 completed the PSQI alone, and 106 completed the STOP-BANG alone. A total of 958 NAFLD participants were eligible for inclusion. Due to incomplete questionnaire responses, analyses involving PSQI and STOP-BANG scores were conducted using available-case data.

The questionnaires used in the study included the Pittsburgh Sleep Quality Index (PSQI) and the STOPBANG questionnaire:

1. Sleep quality was assessed using the Pittsburgh Sleep Quality Index (PSQI), a validated 19-item self-report questionnaire evaluating sleep quality over the preceding four weeks across seven components: subjective sleep quality, sleep latency, sleep duration, habitual sleep efficiency, sleep disturbances, use of sleeping medication, and daytime dysfunction. Each component is scored from 0 to 3, yielding a global score ranging from 0 to 21; a global PSQI score ≥ 6 was used to define poor sleep quality (sleep disturbance) [16]. The reliability and validity of the Persian version of the PSQI have been confirmed, with a Cronbach’s alpha of 0.81 [17]. In this study, PSQI scores of 6–10, 11–15, and > 15 was considered indicative mild, moderate, and severe disturbance, respectively.
2. Obstructive sleep apnea (OSA) risk was assessed using the STOP-BANG questionnaire, an 8-item validated screening tool evaluating snoring, tiredness, observed apnea, blood pressure, BMI > 35, age > 50, neck circumference > 40 cm, and male gender [18, 19]. Each affirmative response scores one point; total scores range from 0 to 8. Participants were classified as low risk (0–2), intermediate risk ([3–4]), or high risk [5–8] for OSA [19, 20]. The Persian version of the STOP-BANG has been validated for use in epidemiological studies in Iran [21].

STOP-BANG identifies individuals at elevated probability of OSA and is not diagnostic; polysomnography was not performed, and no participant in this analysis has confirmed OSA.

Demographic and clinical data including age, sex, marital status, number of children, history of underlying diseases (diabetes, hypertension, cardiac disease, thyroid disease, hepatitis B), and current medications were extracted from the POCS registry. Anthropometric measurements including weight, height, and neck circumference were obtained by trained personnel using standardized protocols. BMI was calculated as weight in kilograms divided by height in meters squared (kg/m²). Fasting serum triglyceride and LDL cholesterol levels were obtained from the POCS biochemical database. All data were extracted retrospectively from the POCS registry using a pre-designed standardized checklist.

No a priori sample size calculation was performed. All POCS participants meeting the eligibility criteria were included (complete enumeration), and the achieved sample size was therefore determined by cohort composition rather than by a target precision. Post hoc power was not calculated, as it is a deterministic function of the observed p-value and conveys no information beyond it. The study was accordingly not powered to detect small differences between subgroups, and comparisons involving less frequent categories including moderate sleep disturbance, high OSA risk, and comparisons across steatosis grades should be regarded as exploratory.

Continuous variables were assessed for normality using the Kolmogorov-Smirnov test and visual inspection of histograms. As most continuous variables demonstrated non-normal distributions, nonparametric statistical methods were applied. Continuous variables are presented as median (interquartile range [IQR]: 25th–75th percentile), while categorical variables are reported as frequency and percentage. Mean ± standard deviation (SD) values are additionally provided for descriptive purposes in the overall sample characteristics.

Group comparisons between participants with and without sleep disturbance were performed using the Mann-Whitney U test for continuous variables and the Pearson chi-square test or Fisher’s exact test for categorical variables, as appropriate. Fisher’s exact test was used when expected cell counts were less than 5. Correlations between PSQI scores, STOP-BANG scores, and continuous clinical variables were assessed using Spearman’s rank correlation coefficient. A two-sided p-value < 0.05 was considered statistically significant. All analyses were unadjusted; no multivariable modelling was performed.

This study was approved by the Institutional Ethics Committee of Mashhad University of Medical Sciences (Ethics code: IR.MUMS.MEDICAL.REC.1401.627; approval date: 2023/01/24). The study was conducted in accordance with the Declaration of Helsinki. As this was a retrospective analysis of anonymized registry data, individual informed consent was waived by the Ethics Committee. All patient data were anonymized prior to analysis to ensure confidentiality.

During the preparation of this manuscript, the authors used Claude (Anthropic) to assist with language editing, improving readability, and refining the phrasing of the English text. The tool was not used to generate, analyze, or interpret study data, nor to produce scientific content or conclusions. All output was reviewed and edited by the authors, who take full responsibility for the integrity and accuracy of the content of this manuscript.

This study is reported in accordance with the Strengthening the Reporting of Observational Studies in Epidemiology (STROBE) statement for cross-sectional studies. A completed STROBE checklist is provided as Supplementary File 1.

## Results

A total of 958 patients with NAFLD were included. The majority were male (69.6%), with a mean age of 46.73 ± 9.08 years. Most participants were married (82.8%). The most common comorbidity was hypertension (15.4%), followed by diabetes mellitus (12.3%). Nearly half of participants had grade 2 hepatic steatosis (47.9%), while 38.9% had grade 1 and 13.2% had grade 3. The demographic, anthropometric and laboratory characteristics of the study population are summarized in Table 1.

**Table 1:** Demographic, anthropometric, and laboratory characteristics of NAFLD patients

Characteristic		Frequency (%) or Mean ± SD
Sex	Male	667 (69.6%)
	Female	291 (30.4%)
Age (years)		46.73 ± 9.08
Height (cm)		170.45 ± 9.67
Weight (kg)		82.13 ± 12.95
BMI (kg/m²)		28.23 ± 7.22
Neck Circumference (cm)		38.32 ± 3.89
Marital Status	Single	164 (17.2%)
	Married	789 (82.8%)
Previous Medical History	Diabetes Mellitus	115 (12.3%)
	Hypertension	144 (15.4%)
	Myocardial Infarction	15 (1.6%)
	Cerebrovascular Accident	1 (0.1%)
	Hepatitis B	3 (0.3%)
	Thyroid Disease	71 (7.4%)
Current Medications	Anti-diabetic agents	100 (10.4%)
	Anti-hypertensive agents	40 (4.2%)
	Lipid-lowering agents	67 (7.0%)

Data presented as frequency (%) for categorical variables and mean ± SD for continuous variables

Table 2 demonstrates nearly half of all participants (47.9%, *n* = 461) had grade 2 (moderate) hepatic steatosis, while 38.9% (*n* = 373) had grade 1 and 13.2% (*n* = 124) had grade 3.

**Table 2:** Distribution of hepatic steatosis grades in NAFLD patients (*n* = 958)

Hepatic Steatosis Grade	Number of Patients (*n*)	Percentage (%)	Total (*n*)
Grade 1 (Mild)	373	38.9%	**958**
Grade 2 (Moderate)	461	47.9%	
Grade 3 (Severe)	124	13.2%	

NAFLD graded by abdominal ultrasonography: Grade 1 (mild), Grade 2 (moderate), Grade 3 (severe)

Complete PSQI data were available for 852 participants. Table 3 represents the sleep status of the participants based on their scores on the PSQI questionnaire. Based on PSQI scores, 302 patients (35.4%) had sleep disturbance, while 550 (64.6%) had normal sleep quality. Of these, 269 (31.6%) had mild disturbance (scores 6–10) and 33 (3.8%) had moderate disturbance (scores 11–15). No patient had severe sleep disturbance (score > 15). The average score of the participants in this questionnaire was 5.06 ± 2.40.

**Table 3:** Sleep quality status of NAFLD patients based on PSQI scores

Sleep Status Category	PSQI Score Range	Number of Patients (*n*)	Percentage (%)
No Sleep Disturbance	≤ 5	550	64.6%
Mild Disturbance	6–10	269	31.6%
Moderate Disturbance	11–15	33	3.8%
Severe Disturbance	> 15	0	0.0%
**TOTAL**		**852**	**100.0%**

Mean PSQI score = 5.06 ± 2.40. PSQI ≥ 6 defines poor sleep quality (sleep disturbance). Total with complete PSQI data = 852

According to STOP-BANG scores (completed by 877 participants), 221 patients (25.2%) were at low risk, 468 (53.4%) at intermediate risk, and 188 (21.4%) at high risk for OSA. The mean STOP-BANG score was 3.43 ± 1.37 (Table 4).

**Table 4:** Distribution of OSA risk categories based on STOP-BANG screening scores

OSA Risk Category	STOP-BANG Score Range	Number of Patients (*n*)	Percentage (%)
Low Risk	0–2	221	25.2%
Intermediate Risk	3–4	468	53.4%
High Risk	5–8	188	21.4%
**TOTAL**		**877**	**100.0%**

Mean STOP-BANG score = 3.43 ± 1.37. Low risk (0–2): no/low probability of OSA; Intermediate risk [3–4]: moderate probability; High risk [5–8]: high probability. Total with complete STOP-BANG data = 877

Of the variables examined, four showed statistically significant differences between patients with and without sleep disturbance: sex, marital status, BMI, and hypertension (Table 5).

Sex was significantly associated with sleep disturbance (*p* = 0.022). Poor sleep quality was present in 101 of 244 women (41.4%) compared with 201 of 608 men (33.1%), a difference of approximately 8% points. Marital status also showed a significant association (*p* = 0.019): sleep disturbance was present in 66 of 150 single patients (44.0%) compared with 235 of 697 married patients (33.7%).

Regarding anthropometric measures, BMI was significantly higher in patients with sleep disturbance compared to those without [median 28.16 (IQR: 25.61–30.60) vs. 27.53 (IQR: 25.58–29.68) kg/m²; *p* = 0.026]. In contrast, no significant differences were observed between the two groups in terms of age [47 (IQR: 40–53) vs. 45.5 (IQR: 39–53) years; *p* = 0.197], height [172 (IQR: 166–177) vs. 171 (IQR: 164–177) cm; *p* = 0.073], weight [81.6 (IQR: 73.6–89.6) vs. 81.5 (IQR: 71.9–92.4) kg; *p* = 0.681], or neck circumference [39 (IQR: 37–41) vs. 39 (IQR: 36–42) cm; *p* = 0.642].

Among comorbidities, hypertension was the only condition that showed a statistically significant association with sleep disturbance, being more prevalent in patients with sleep disturbance (19.8%) than in those without (12.7%; *p* = 0.007). Other comorbidities, including diabetes mellitus (11.7% vs. 11.8%; *p* = 0.999), myocardial infarction (2.0% vs. 1.5%; *p* = 0.580), cerebrovascular accident (0.0% vs. 0.2%; *p* > 0.999), hepatitis B (0.7% vs. 0.0%; *p* = 0.125), and thyroid disease (9.7% vs. 6.6%; *p* = 0.137), did not differ significantly between the two groups.

With respect to current medications, no significant differences were found between patients with and without sleep disturbance in the use of anti-diabetic agents (10.9% vs. 10.4%; *p* = 0.817), anti-hypertensive agents (5.6% vs. 3.1%; *p* = 0.098), or lipid-lowering agents (7.6% vs. 5.8%; *p* = 0.311).

Finally, laboratory parameters including serum triglycerides [147 (IQR: 94–188) vs. 142 (IQR: 95–198) mg/dL; *p* = 0.523] and serum LDL-cholesterol [139 (IQR: 119–164) vs. 142 (IQR: 119–170) mg/dL; *p* = 0.086] were comparable between the two groups, with no statistically significant differences observed.

**Table 5:** Comparison of characteristics between NAFLD patients with and without sleep disturbance

Characteristic		Without Sleep Disturbance *n* (%)	With Sleep Disturbance *n* (%)	*P*
Sex	Male	407 (74.0%)	201 (66.6%)	0.022 ᵃ
	Female	143 (26.0%)	101 (33.4%)	
Age (years)		47 (40–53)	45.5 (39–53)	0.197 ᵇ
Height (cm)		172 (166–177)	171 (164–177)	0.073 ᵇ
Weight (kg)		81.6 (73.6–89.6)	81.5 (71.9–92.4)	0.681 ᵇ
BMI (kg/m²)		27.53 (25.58–29.68)	28.16 (25.61–30.60)	0.026 ᵇ
Neck Circumference (cm)		39 (37–41)	39 (36–42)	0.642 ᵇ
Marital Status	Single	84 (15.4%)	66 (21.9%)	0.019 ᵃ
	Married	462 (84.6%)	235 (78.1%)	
Previous Medical History	Diabetes Mellitus	64 (11.8%)	35 (11.7%)	0.999 ᵃ
	Hypertension	69 (12.7%)	59 (19.8%)	0.007 ᵃ
	Myocardial Infarction	8 (1.5%)	6 (2.0%)	0.580 ᵃ
	Cerebrovascular Accident	1 (0.2%)	0 (0.0%)	> 0.999 ᶜ
	Hepatitis B	0 (0.0%)	2 (0.7%)	0.125 ᶜ
	Thyroid Disease	36 (6.6%)	29 (9.7%)	0.137 ᵃ
Current Medications	Anti-diabetic agents	57 (10.4%)	33 (10.9%)	0.817 ᵃ
	Anti-hypertensive agents	17 (3.1%)	17 (5.6%)	0.098 ᵃ
	Lipid-lowering agents	32 (5.8%)	23 (7.6%)	0.311 ᵃ
Serum Triglycerides (mg/dL)		142 (95–198)	147 (94–188)	0.523 ᵇ
Serum LDL-Cholesterol (mg/dL)		142 (119–170)	139 (119–164)	0.086 ᵇ

Continuous variables presented as median (IQR: 25th–75th percentile). Percentages are column percentages, calculated within each sleep-disturbance group. Analyses were conducted on an available-case basis, and denominators therefore vary between variables: sex and medication data were available for 852 participants (550 without and 302 with sleep disturbance), marital status for 847 (546 and 301), and previous medical history for 841 (543 and 298). ᵃ Chi-square test. ᵇ Mann-Whitney U test. ᶜ Fisher’s exact test

Sleep disturbance prevalence was 33.0% in grade 1, 29.9% in grade 2, and 33.1% in grade 3, showing no consistent trend across steatosis severity grades. Chi-square analysis revealed no statistically significant association between hepatic steatosis grade and the presence of sleep disturbance (*p* = 0.193; Table 6). These findings indicate that no notable association was observed between steatosis grade and sleep disturbance.

**Table 6:** Association between hepatic steatosis grade and sleep disturbance

Hepatic Steatosis Grade	Total Patients (*n*)	Without Sleep Disturbance	With Sleep Disturbance	Sleep Disturbance Rate (%)
Grade 1 (Mild)	326	203	123	33.0%
Grade 2 (Moderate)	424	286	138	29.9%
Grade 3 (Severe)	102	61	41	33.1%
**TOTAL**	**852**	**550**	**302**	**35.4%**

*p* = 0.193 (Chi-square test)

A significant association was found between OSA risk category and sleep disturbance (*p* = 0.009). Among patients with sleep disturbance, 26.4% were at high OSA risk compared to 18.1% of those without sleep disturbance (Table 7).

**Table 7:** Association between obstructive sleep apnea risk (STOP-BANG) and sleep disturbance in NAFLD patients

OSA Risk Category	Without Sleep Disturbance *n* (%)	With Sleep Disturbance *n* (%)	*P*-value
Low Risk (STOP-BANG 0–2)	156 (28.8%)	65 (22.3%)	0.009 ᵃ
Intermediate Risk (STOP-BANG 3–4)	287 (53.0%)	150 (51.4%)	
High Risk (STOP-BANG 5–8)	98 (18.1%)	77 (26.4%)	

ᵃ Chi-square test

Spearman’s correlation analysis revealed a statistically significant, albeit weak, positive correlation between BMI and PSQI global score (*r* = 0.072, *p* = 0.036). Additionally, PSQI score was significantly positively correlated with STOP-BANG score (*r* = 0.168, *p* < 0.001). No significant correlations were found between PSQI scores and age, height, weight, neck circumference, serum triglycerides, or LDL cholesterol (all *p* > 0.05). The full set of correlations between global PSQI score and the anthropometric and laboratory variables is presented in Table 8.

**Table 8:** Spearman’s correlation between PSQI global score and anthropometric/laboratory variables in NAFLD patients

Variable	Spearman’s *r*	*P*-value
Age	−0.055	0.111
Height (cm)	−0.049	0.151
Weight (kg)	0.017	0.618
BMI (kg/m²)	0.072	0.036*
Neck Circumference (cm)	0.002	0.947
Serum Triglycerides (mg/dL)	−0.013	0.709
Serum LDL-Cholesterol (mg/dL)	−0.065	0.056
STOP-BANG Score	0.168	< 0.001*

* Statistically significant (p < 0.05). Spearman's rank correlation coefficient used for all analyses.

STOP-BANG scores showed significant positive correlations with all anthropometric and biochemical variables examined: age (*r* = 0.154, *p* < 0.001), height (*r* = 0.314, *p* < 0.001), weight (*r* = 0.385, *p* < 0.001), BMI (*r* = 0.201, *p* < 0.001), neck circumference (*r* = 0.510, *p* < 0.001), serum triglycerides (*r* = 0.165, *p* < 0.001), LDL cholesterol (*r* = 0.116, *p* = 0.001), and hepatic steatosis grade (*r* = 0.232, *p* < 0.001). These correlations are presented in Table 9.

**Table 9:** Spearman’s correlation between STOP-BANG score and anthropometric/laboratory variables in NAFLD patients

Variable	Spearman’s *r*	*P*-value
Age	0.154	< 0.001*
Height (cm)	0.314	< 0.001*
Weight (kg)	0.385	< 0.001*
BMI (kg/m²)	0.201	< 0.001*
Neck Circumference (cm)	0.510	< 0.001*
Serum Triglycerides (mg/dL)	0.165	< 0.001*
Serum LDL-Cholesterol (mg/dL)	0.116	0.001*
Hepatic Steatosis Grade	0.232	< 0.001*

* Statistically significant ( *p *< 0.05). Spearman’s rank correlation coefficient used for all analyses. Age, BMI and neck circumference are constituent items of the STOP-BANG questionnaire, and height and weight contribute to it through BMI; their correlations with the total score are therefore structurally determined and should not be interpreted as empirical associations. Serum triglycerides, LDL-cholesterol and hepatic steatosis grade are not components of the instrument, and their correlations are interpretable

## Discussion

The present cross-sectional study provides, to our knowledge, one of the largest single-cohort assessments of sleep quality and obstructive sleep apnea (OSA) risk in Iranian patients with non-alcoholic fatty liver disease (NAFLD). Drawing on prospectively collected data from the Persian Organizational Cohort Study (POCS) at Mashhad University of Medical Sciences, we evaluated 958 NAFLD patients using two validated instruments (PSQI and the STOP-BANG questionnaire) and demonstrated that sleep disturbance is a prevalent and clinically meaningful comorbidity in this population. Specifically, 35.4% of patients with available PSQI data met the threshold for poor sleep quality (global score ≥ 6), with the large majority falling in the mild disturbance category (31.6%) and a smaller but notable proportion classified as moderate (3.8%). Notably, no patient in our cohort exhibited severe sleep disturbance, a pattern that may reflect the relatively younger age profile of our sample (mean age 46.7 years) or the exclusion of patients on regular sedative medications, who might otherwise anchor the upper end of the PSQI score distribution. Simultaneously, more than half of participants (53.4%) were at intermediate risk for OSA based on STOP-BANG scores, and approximately one in five (21.4%) were at high risk, underscoring a substantial and largely unscreened burden of sleep-disordered breathing in this cohort.

The overall prevalence of sleep disturbance observed in our study is broadly consistent with, though somewhat lower than, figures reported in comparable cohorts. In a large Japanese cross-sectional survey of 4,828 participants, Takahashi et al. identified significant associations between multiple PSQI components (particularly daytime dysfunction) and NAFLD, with notable sex-specific patterns [12]. A population-based Taiwanese study by Chou et al., enrolling 6,663 adults with ultrasonographic assessment of hepatic steatosis, produced a more subtle and in some respects counterintuitive view: after adjustment for potential confounders, poor sleep quality (as defined by a PSQI score greater than 5) was inversely associated with mild and moderate-to-severe NAFLD in males, while sleep duration was not independently associated with NAFLD. In women, sleep condition was not associated with NAFLD [22]. In a recent meta-analysis of 15 studies encompassing 261,554 participants, short sleep duration was found to confer a statistically notable increase in NAFLD risk (OR 1.15; 95% CI 1.04–1.28), further supporting the idea that aberrant sleep patterns and hepatic steatosis are meaningfully intertwined [9]. Of particular relevance to our study is work from another Iranian PERSIAN cohort site (the Shahrekord cohort) in which Zarean et al. analyzed 9,151 adults including 1,320 with NAFLD and found that NAFLD was associated with shorter sleep duration, worse sleep efficiency, and more frequent use of sleeping pills, with the strongest associations observed in older age groups [23]. The convergence between our Mashhad cohort findings and those of the Shahrekord PERSIAN cohort, despite differences in geographic location, sample composition, and study protocol, adds meaningful intra-national consistency to the evidence that sleep disturbance is a prevalent and clinically relevant companion to NAFLD in the Iranian population. The somewhat lower overall prevalence of poor sleep quality in our sample compared with some reported cohorts may in part reflect the exclusion of patients on regular sedative or hypnotic medications (a group likely to carry a disproportionate burden of sleep pathology) as well as differences in the age structure and sex distribution of enrolled participants.

One of the more clinically informative findings of this study was the significant association between female sex and sleep disturbance among NAFLD patients (*p* = 0.022). Poor sleep quality was present in 41.4% of women compared with 33.1% of men, a difference of practical relevance given that women already represent a minority of our cohort (30.4% overall). For instance, in an Iranian case-control study analysis revealed that poor sleep quality is significantly more common in women than in men [14]. In a cross-sectional survey of 4,828 Japanese health check-up participants, Takahashi et al. found that daytime dysfunction was significantly associated with NAFLD in both men (OR 2.82, 95% CI 1.39–5.75) and women (OR 2.08, 95% CI 1.10–3.92); however, after adjusting for BMI, the predictive PSQI component diverged by sex and sleep latency emerged as the significant predictor in men, while daytime dysfunction persisted as the relevant component in women. Demonstrating that the sleep-NAFLD relationship operates through sex-specific pathways rather than a uniform mechanism [12]. Complementarily, Chou et al., in a Taiwanese general population study, found that poor sleep quality was associated with NAFLD only in males and that no significant sleep-NAFLD association was detectable in females after confounder adjustment, further reinforcing the biological heterogeneity of this relationship across sexes [22]. The higher prevalence of sleep disturbance among women in our cohort may partly reflect the well-documented tendency for Iranian women to report poorer sleep quality compared with Iranian men in population-based studies [24], suggesting that the female sex effect observed here is not unique to NAFLD but rather mirrors a broader epidemiological pattern in this population. These associations are unadjusted. Female sex, single marital status, BMI and hypertension are strongly interrelated in this population women differ from men in BMI distribution, and marital status is associated with both age and sex and the present analysis cannot determine whether any one of these associations is independent of the others. They should therefore be understood as identifying characteristics that co-occur with poor sleep quality, rather than as independent determinants of it.

BMI was significantly higher in patients with sleep disturbance compared to those without [median 28.16 (IQR 25.61–30.60) vs. 27.53 (IQR 25.58–29.68) kg/m², *p* = 0.026]; however, Spearman’s correlation between BMI and global PSQI score, while statistically significant, was negligible (*r* = 0.072, *p* = 0.036), with BMI accounting for less than 1% of individual-level PSQI variance (r²<0.01). This weak magnitude, consistent with correlations reported in comparable adult populations [25], reflects the multidetermined nature of sleep disturbance in NAFLD. a finding further supported by meta-analytic evidence confirming that the adiposity–sleep association is weak [26]. Clinically, these data argue against restricting sleep quality screening to the most obese NAFLD patients, as individuals across the full BMI spectrum may carry a significant and otherwise undetected sleep burden.

Marital status was significantly associated with sleep disturbance, with single patients more frequently affected than married counterparts (21.9% vs. 15.4%; *p* = 0.019). This is consistent with meta-analytic evidence demonstrating a moderate positive correlation between couple relationship quality and sleep quality (*r* = 0.34) and sleep duration (*r* = 0.39) across 62 studies and 43,860 participants [27], and with longitudinal data showing that transitioning into marriage independently improves sleep quality compared to remaining single or divorced [28]. a point that warrants consideration in future studies examining social determinants of sleep in NAFLD populations.

Among the comorbidities examined, hypertension was the only condition significantly associated with sleep disturbance (19.8% vs. 12.7%; *p* = 0.007). This reflects a well-characterized bidirectional relationship: sleep disruption activates the sympathetic nervous system and attenuates nocturnal blood pressure dipping, while hypertension reciprocally impairs sleep through nocturnal arousal and polyuria [29, 30], with meta-analytic evidence confirming short sleep duration as an risk factor for incident hypertension [31]. For clinicians managing NAFLD patients, the co-existence of hypertension and sleep disturbance in the same individual is a practical signal to look beyond the liver and assess the patient’s broader cardiometabolic risk.

The absence of significant associations with other comorbidities does not necessarily mean they are unrelated to sleep disturbance in NAFLD patients. Both type 2 diabetes and thyroid dysfunction have well-established links to poor sleep quality in the broader literature [32–34], and the fact that we did not detect these associations likely reflects the relatively small number of affected patients in our cohort rather than a genuine lack of biological relationship, a limitation that adequately powered future studies should address.

We found no statistically significant association between hepatic steatosis grade and sleep disturbance prevalence (*p* = 0.193), with rates remaining flat across all three grades. This negative finding should be interpreted narrowly. Ultrasonographic grading reflects the extent of hepatic fat accumulation and correlates only loosely with necroinflammatory activity and fibrosis stage, which are the dimensions of liver disease most plausibly linked to systemic and sleep-related pathophysiology; in a prospective study of patients with biopsy-proven NAFLD, no radiological modality including ultrasonography detected hepatocyte ballooning, Mallory’s hyaline or fibrosis, and only steatosis severity was reflected in imaging findings [35]. Our data therefore indicate an absence of association with steatosis burden, not with liver disease severity; participants with advanced fibrosis, whom our imaging could not identify, may exhibit a different relationship. This distinction may account for the divergence from Bernsmeier et al., who, working with biopsy-proven NAFLD, demonstrated that sleep disturbance correlated significantly with ALT (*r* = 0.44, *p* = 0.003), AST (*r* = 0.46, *p* = 0.002), and fibrosis stage (*r* = 0.364, *p* = 0.019) [36]. Their measures captured hepatocellular injury and fibrosis, whereas ours captured steatosis burden alone; the two studies may therefore be examining different aspects of liver disease rather than producing genuinely conflicting results, particularly given evidence of a positive relationship between transaminase levels and ultrasonographic steatosis grade [37]. Notably, OSA risk by STOP-BANG did correlate with steatosis grade (*r* = 0.232, *p* < 0.001), consistent with population-based evidence linking higher STOP-BANG scores to increasing NAFLD prevalence [38] and meta-analytic data showing OSA severity independently correlates with ALT elevation and histological NAFLD progression [39]. These observations suggest that intermittent hypoxia, rather than subjective sleep quality, may be the sleep-related mechanism most closely tied to hepatic disease severity.

The prevalence of intermediate and high OSA risk in our cohort were 53.4% and 21.4%, respectively, based on STOP-BANG scores. The mean STOP-BANG score of 3.43 ± 1.37 placed the average participant in the intermediate-risk category. A significant association was found between higher OSA risk category and the presence of sleep disturbance (*p* = 0.009), with 26.4% of sleep-disturbed patients at high OSA risk compared with only 18.1% of those without sleep disturbance, and STOP-BANG score correlated **weakly** with global PSQI score (*r* = 0.168, *p* < 0.001), the two instruments sharing approximately 3% of their variance. Furthermore, an important caveat applies to several of these correlations. Age, BMI and neck circumference are constituent items of the STOP-BANG questionnaire, and height and weight contribute to it through BMI; their positive correlations with the total score are mathematically expected and carry no independent evidential weight. The correlation with neck circumference (*r* = 0.510), the strongest in our data, is the clearest illustration of this and reflects the construction of the instrument rather than a property of our population. By contrast, serum triglycerides (*r* = 0.165), LDL-cholesterol (*r* = 0.116) and hepatic steatosis grade (*r* = 0.232) are not components of STOP-BANG, and their correlations with it are interpretable though modest in magnitude and it is these that support a systemic metabolic dimension to OSA risk in this cohort. In a meta-analysis of nine studies comprising 2,272 participants, Jin et al. demonstrated that OSA was independently associated with hepatic steatosis, lobular inflammation, and fibrosis, and that OSA severity correlated with serum ALT levels [39]. Trzepizur et al. demonstrated in a multicenter cohort that in patients with metabolic comorbidities severe OSA has been independently associated with increased liver stiffness as measured by transient elastography, suggesting a gradient of hepatic fibrotic risk with severity of OSA [40]. In another study using blood marker of liver steatosis (the hepatic steatosis index), it was determined the risk of liver steatosis increased with the severity of OSA and sleep-related hypoxemia [41]. Most recently, Trzepizur et al. in 2026 study provided the strongest evidence to date that OSA is independently associated with advanced liver fibrosis in biopsy-proven NAFLD patients [42]. The modest but consistent correlation between STOP-BANG score and steatosis grade in our cohort (*r* = 0.232, *p* < 0.001), corresponding to approximately 5% of shared variance, is consistent with the findings of Kim et al., who demonstrated in 4,275 nationally representative Korean adults that NAFLD prevalence increased significantly with higher STOP-BANG scores in both sexes, with the association being more pronounced in obese individuals [38]. Benotti et al., studying 362 bariatric surgery patients with biopsy-proven NAFLD and polysomnography-confirmed OSA, demonstrated: [1] OSA severity (measured by apnea-hypopnea index) has positive correlation with severity of NAFLD [2] bidirectional screening of OSA patients for NAFLD and of NAFLD patients for OSA is clinically warranted regardless of whether metabolic syndrome is present or absent [43]. Taken together, despite relying on STOP-BANG screening rather than polysomnography, and lacking liver enzyme and histological data, our findings tell a coherent story: OSA risk in NAFLD patients is not simply a sleep issue, it reflects the same systemic metabolic burden that drives steatosis progression, and it can be meaningfully captured even with a simple questionnaire.

Our data suggest a pragmatic, tiered approach. Given that more than one third of participants met the threshold for poor sleep quality and that sleep disturbance was distributed across the full BMI range rather than concentrated in the most obese, brief questionnaire-based screening with the PSQI and STOP-BANG appears reasonable at the point of NAFLD diagnosis rather than being reserved for selected patients. Referral for polysomnography, however, is unlikely to be justified for all patients; prioritizing those at high STOP-BANG risk (score 5–8), who represented 21.4% of our cohort, would target confirmatory testing towards those with the highest pre-test probability. Patients at intermediate risk (53.4%) may warrant reassessment if symptomatic or if metabolic control deteriorates. We emphasize that this pathway is a reasoned extrapolation from cross-sectional screening data and has not been prospectively validated; its yield and cost-effectiveness in NAFLD populations remain to be established.

Cardiovascular disease is the leading cause of death in NAFLD, so coexisting OSA risk carries cardiometabolic relevance [44]. Intermittent hypoxemia and sympathetic activation promote atrial remodeling; among patients undergoing atrial flutter ablation, obstructive sleep apnea was independently associated with long-term atrial fibrillation [45]. Obesity mechanistically links the two conditions: BMI and neck circumference are STOP-BANG items because adiposity narrows the upper airway, and a 10% weight gain predicts a six-fold rise in moderate-to-severe sleep-disordered breathing [46, 47]. Obesity has been linked to lower mortality after acute coronary syndrome [48], yet this reverses when central adiposity is measured, normal BMI with central obesity carrying the worst survival [49]. The paradox therefore appears measurement-dependent rather than protective. Management should therefore be holistic, incorporating weight control, physical activity and sleep assessment [50, 51].

Strengths and Limitations.

This study benefits from a large, well-characterized clinical cohort (*n* = 958), use of two validated instruments (PSQI and STOP-BANG), and standardized ultrasonographic grading of hepatic steatosis, enhancing internal consistency and reproducibility.

Several limitations should be emphasized. First, OSA risk was assessed by questionnaire; polysomnography, the reference standard, was not performed, and our estimates therefore describe screening-defined risk rather than disease prevalence. Second, hepatic assessment was limited to ultrasonographic steatosis grading; serum transaminases, non-invasive fibrosis markers and histology were unavailable, so we could not examine whether sleep characteristics relate to necro inflammatory activity or fibrosis stage. Third, measures of insulin resistance were not available, precluding assessment of the metabolic pathway most plausibly linking sleep disturbance to steatosis. Fourth, all reported associations are unadjusted; the variables required for credible multivariable modelling were not present in the analytic dataset, and the reported associations may therefore reflect confounding by interrelated factors including adiposity, sex, age and hypertension. Fifth, Analyses were conducted on an available-case basis, with PSQI data missing for 106 participants (11.1%) and STOP-BANG data for 81 (8.5%). We could not assume these data were missing completely at random; if non-response was related to sleep complaints, comorbidity burden or health literacy, the reported prevalence estimates may be biased, and the direction of such bias cannot be determined from the available data. Sixth, Sleep quality was analyzed using the global PSQI score; component-level scores were not available in the analytic dataset. Because the seven PSQI components capture distinct aspects of sleep and may relate differently to hepatic steatosis, the global score may obscure domain-specific associations. Component-level analysis represents an important direction for subsequent work in this cohort. Seventh, our exclusion criteria did not encompass viral hepatitis. Three participants (0.3%) had a recorded history of hepatitis B and were retained in the analysis. Although coexisting hepatitis B infection may contribute to hepatic steatosis and complicates its attribution to metabolic causes, the number of affected participants is too small to have materially influenced any of the estimates reported here.

Our findings derive from an organizational cohort of university employees aged 30–70 years, a population with above-average health literacy and occupational stability, and with a marked male predominance. The absence of severe sleep disturbance in this sample may reflect these characteristics. Generalization to the wider Iranian population, to older adults, or to clinically referred NAFLD patients should therefore be made with caution.

## Conclusion

Because the present analysis is cross-sectional, the temporal ordering of sleep disturbance and hepatic steatosis cannot be determined, and no causal inference is possible. Our findings identify sleep disturbance and elevated OSA risk as frequent co-occurring conditions in NAFLD that merit further evaluation in prospective studies, rather than as modifiable targets demonstrated to improve metabolic outcomes.

## Supplementary Information

Below is the link to the electronic supplementary material.

Supplementary Material 1

## Data Availability

The datasets analyzed during the current study are not publicly available because they are part of the PERSIAN Organizational Cohort Study (POCS) at Mashhad University of Medical Sciences and are subject to institutional and cohort governance restrictions, but are available from the corresponding author on reasonable request and with the permission of the POCS/PERSIAN cohort administration at Mashhad University of Medical Sciences.

## Abbreviations

AHI: Apnea/Hypopnea Index
ALT: Alanine Aminotransferase
BMI: Body Mass Index
HPA: Hypothalamic–Pituitary–Adrenal
IQR: Interquartile Range
LDL: Low-Density Lipoprotein
MAFLD: Metabolic-Associated Fatty Liver Disease
MASH: Metabolic Dysfunction-Associated Steatohepatitis
MASLD: Metabolic Dysfunction-Associated Steatotic Liver Disease
MUMS: Mashhad University of Medical Sciences
NAFLD: Non-Alcoholic Fatty Liver Disease
NASH: Non-Alcoholic Steatohepatitis
NSW: Night Shift Work
OHS: Obesity Hypoventilation Syndrome
OSA: Obstructive Sleep Apnea
POCS: PERSIAN Organizational Cohort Study
PSQI: Pittsburgh Sleep Quality Index
REM: Rapid Eye Movement
SD: Standard Deviation
STOP-BANG: Snoring, Tiredness, Observed apnea, blood Pressure, BMI, Age, Neck circumference, Gender
