# Supplementary material for: Evaluation of sleep quality and obstructive sleep apnea in non-alcoholic fatty liver disease: a cross-sectional study
Source: BMC Gastroenterol. 2026 Sep 2;26:583. doi: 10.1186/s12876-026-05298-z (PMC13595775; doi:10.1186/s12876-026-05298-z)
Supplement: Supplementary file 1 [file 12876_2026_5298_MOESM1_ESM.docx]

**STROBE Statement — Checklist of items that should be included in reports of cross-sectional studies**

*Evaluation of Sleep Quality and Obstructive Sleep Apnea in Non-Alcoholic Fatty Liver Disease: A Cross-Sectional Study*

Submitted to BMC Gastroenterology • Supplementary File 1 • Manuscript ID: 9947ba80-9814-434a-b8eb-f7425254706b

| **Section / Topic** | **Item** | **Recommendation** | **Reported in / Comment** |
| --- | --- | --- | --- |
| **Title and Abstract** | | | |
| Title and abstract | 1 (a) | Indicate the study’s design with a commonly used term in the title or the abstract | Title. The design is stated as “A Cross-Sectional Study”, and repeated in the first line of the Abstract Methods. |
|  | 1 (b) | Provide in the abstract an informative and balanced summary of what was done and what was found | Abstract (Background, Methods, Results, Conclusions). The Conclusions state that associations were unadjusted and that causal inference is not possible. |
| **Introduction** | | | |
| Background / rationale | 2 | Explain the scientific background and rationale for the investigation being reported | Background, paragraphs 1–6. |
| Objectives | 3 | State specific objectives, including any prespecified hypotheses | Background, final paragraph. Objectives were descriptive; no prespecified hypotheses were formulated. |
| **Methods** | | | |
| Study design | 4 | Present key elements of study design early in the paper | Methods, first sentence: cross-sectional analysis of baseline data from the PERSIAN Organizational Cohort Study (POCS). |
| Setting | 5 | Describe the setting, locations, and relevant dates, including periods of recruitment, exposure, follow-up, and data collection | Methods, first paragraph. Setting: POCS at Mashhad University of Medical Sciences, Iran. Baseline enrolment began after ethics approval in January 2017 and was completed in mid-2021; data were extracted from the cohort registry on 4 July 2023. |
| Participants | 6 (a) | Give the eligibility criteria, and the sources and methods of selection of participants | Methods, paragraphs 2–3, and Figure 1. Eligible participants had ultrasonographically confirmed NAFLD and completed at least one of the PSQI or STOP-BANG. Five exclusion criteria are listed. Selection was by complete enumeration of the cohort. |
| Variables | 7 | Clearly define all outcomes, exposures, predictors, potential confounders, and effect modifiers. Give diagnostic criteria, if applicable | Methods. Hepatic steatosis graded 1–3 by defined ultrasonographic criteria; poor sleep quality defined as global PSQI ≥ 6; OSA risk categorised as low (0–2), intermediate (3–4) or high (5–8) on STOP-BANG. No confounders were adjusted for; this is stated explicitly. |
| Data sources / measurement | 8 | For each variable of interest, give sources of data and details of methods of assessment (measurement). Describe comparability of assessment methods if there is more than one group | Methods. Demographic, clinical and biochemical data extracted from the POCS registry using a standardised checklist; anthropometry by trained personnel using standardised protocols; Persian-validated PSQI and STOP-BANG instruments. Assessment methods were identical for all participants. |
| Bias | 9 | Describe any efforts to address potential sources of bias | Methods (standardized instruments, uniform registry-based data extraction, pre-specified exclusion criteria) and Limitations, where potential selection bias arising from questionnaire non-response is discussed and its direction acknowledged as indeterminate. |
| Study size | 10 | Explain how the study size was arrived at | Methods (Statistical Analysis). No a priori sample size calculation was performed; a census (complete enumeration) approach was used and the achieved size was determined by cohort composition. Post hoc power was not calculated, with the reason given. |
| Quantitative variables | 11 | Explain how quantitative variables were handled in the analyses. If applicable, describe which groupings were chosen and why | Methods. PSQI analyzed both as a continuous global score and dichotomized at the validated cut-off of ≥ 6; STOP-BANG analysed continuously and in the three established risk bands. Steatosis analysed by ultrasonographic grade. |
| Statistical methods | 12 (a) | Describe all statistical methods, including those used to control for confounding | Methods (Statistical Analysis). Kolmogorov–Smirnov test for normality; median (IQR) for non-normally distributed variables; Mann–Whitney U; Pearson chi-square and Fisher’s exact tests; Spearman’s rank correlation; two-sided p < 0.05. It is stated that all analyses were unadjusted and that no multivariable modelling was performed. |
|  | 12 (b) | Describe any methods used to examine subgroups and interactions | *Not applicable. No subgroup or interaction analyses were performed. The sex and marital status comparisons presented in Table 5 are univariable chi-square analyses of the whole analysis set, not stratified analyses.* |
|  | 12 (c) | Explain how missing data were addressed | Methods (Statistical Analysis) and Limitations. Analyses were conducted on an available-case basis. Numbers with missing data are reported in Figure 1, in the Limitations, and in the footnote to Table 5. |
|  | 12 (d) | If applicable, describe analytical methods taking account of sampling strategy | *Not applicable. A census of all eligible cohort participants was used; no sampling strategy requiring analytical adjustment was applied.* |
|  | 12 (e) | Describe any sensitivity analyses | *Not applicable. No sensitivity analyses were performed.* |
| **Results** | | | |
| Participants | 13 (a) | Report numbers of individuals at each stage of study—eg numbers potentially eligible, examined for eligibility, confirmed eligible, included in the study, completing follow-up, and analysed | Figure 1 and Methods. 11,023 screened; 9,480 without ultrasonographic steatosis; 1,543 with steatosis; 585 excluded under pre-specified criteria; 958 included; 852 contributed to the PSQI analysis and 877 to the STOP-BANG analysis. |
|  | 13 (b) | Give reasons for non-participation at each stage | Figure 1. Reasons and numbers are given for both exclusion stages, including 153 participants who completed neither questionnaire. |
|  | 13 (c) | Consider use of a flow diagram | Figure 1 (participant flow diagram). |
| Descriptive data | 14 (a) | Give characteristics of study participants (eg demographic, clinical, social) and information on exposures and potential confounders | Tables 1 and 5; Results, first section. |
|  | 14 (b) | Indicate number of participants with missing data for each variable of interest | Figure 1 and footnote to Table 5. Sex and medication data were available for 852 participants, marital status for 847, and previous medical history for 841. PSQI data were missing for 106 participants (11.1%) and STOP-BANG data for 81 (8.5%). |
| Outcome data | 15 | Report numbers of outcome events or summary measures | Tables 2–4. Poor sleep quality in 302 of 852 participants (35.4%); intermediate OSA risk in 53.4% and high OSA risk in 21.4% of 877 participants. |
| Main results | 16 (a) | Give unadjusted estimates and, if applicable, confounder-adjusted estimates and their precision (eg, 95% confidence interval). Make clear which confounders were adjusted for and why they were included | *Tables 5–9. Unadjusted estimates are reported throughout; no confounder-adjusted estimates were calculated, and this is stated in the Methods, Discussion and Limitations. Confidence intervals are not reported; precision limitations are addressed in the Methods (study size) and effect magnitudes are interpreted through shared variance in the Discussion rather than by statistical significance alone.* |
|  | 16 (b) | Report category boundaries when continuous variables were categorized | Methods and Tables 3–4. PSQI bands (0–5, 6–10, 11–15, 16–21) and STOP-BANG bands (0–2, 3–4, 5–8) are stated explicitly. |
|  | 16 (c) | If relevant, consider translating estimates of relative risk into absolute risk for a meaningful time period | *Not applicable. This is a cross-sectional prevalence study; no relative risk estimates over time were calculated.* |
| Other analyses | 17 | Report other analyses done—eg analyses of subgroups and interactions, and sensitivity analyses | *Not applicable. No subgroup, interaction or sensitivity analyses were performed.* |
| **Discussion** | | | |
| Key results | 18 | Summarise key results with reference to study objectives | Discussion, first paragraph, and Conclusion. |
| Limitations | 19 | Discuss limitations of the study, taking into account sources of potential bias or imprecision. Discuss both direction and magnitude of any potential bias | Limitations. Seven limitations are addressed: absence of polysomnography; ultrasonographic assessment only, without transaminases, fibrosis markers or histology; absence of insulin resistance measures; absence of multivariable adjustment; available-case analysis and potential non-random missingness, whose direction is stated to be indeterminate; unavailability of PSQI component scores; and retention of three participants with a history of hepatitis B. |
| Interpretation | 20 | Give a cautious overall interpretation of results considering objectives, limitations, multiplicity of analyses, results from similar studies, and other relevant evidence | Discussion and Conclusion. Associations are described as unadjusted throughout; correlations are interpreted by magnitude and shared variance; correlations between STOP-BANG and its own constituent items are identified as structurally determined; and the negative steatosis finding is interpreted as an absence of association with steatosis burden rather than with liver disease severity. |
| Generalisability | 21 | Discuss the generalisability (external validity) of the study results | Discussion / Limitations. The cohort comprises university employees aged 30–70 years with above-average health literacy and a marked male predominance; generalization to the wider Iranian population, to older adults, and to clinically referred NAFLD patients is stated to require caution. |
| **Other Information** | | | |
| Funding | 22 | Give the source of funding and the role of the funders for the present study and, if applicable, for the original study on which the present article is based | Declarations (Funding). The present study received no specific grant from any funding agency in the public, commercial or not-for-profit sectors. The parent POCS cohort was funded by the Iranian Ministry of Health and Medical Education. |

**Note.** *Items marked “Not applicable” were not performed in this study and are declared as such rather than reported. Item 16 (a) is partially met: unadjusted estimates are reported, but confidence intervals are not; the reasons and the compensating measures are stated in the corresponding row. Page numbers may be added once the manuscript is paginated in its final form.*

An Explanation and Elaboration article discusses each checklist item and gives methodological background and published examples of transparent reporting. The STROBE checklist is best used in conjunction with this article, freely available on the websites of PLoS Medicine, Annals of Internal Medicine and Epidemiology. Information on the STROBE Initiative is available at www.strobe-statement.org.
